# A community perspective on the role of fathers during pregnancy: a qualitative study

**DOI:** 10.1186/1471-2393-13-60

**Published:** 2013-03-07

**Authors:** Amina P Alio, Cindi A Lewis, Kenneth Scarborough, Kenn Harris, Kevin Fiscella

**Affiliations:** 1 Public Health Sciences, University of Rochester School of Medicine & Dentistry, 265 Crittenden Blvd, CU 420644, Rochester, NY 14642, USA; 2 Translational Biomedical Sciences, University of Rochester School of Medicine and Dentistry, 265 Crittenden Blvd CU 420644, Rochester, NY 14642, USA; 3 REACHUP, Incorporated, 2902 N. Armenia Avenue, Suite 100, Tampa, Florida 33607, USA; 4 New Haven Healthy Start, New Haven, Connecticut, 70 Audubon Street, New Haven, Connecticut 06510, USA; 5 Family Medicine, Public Health Sciences, and Oncology, University of Rochester School of Medicine & Dentistry and Wilmot Cancer Center, 1381 South Ave, Rochester, New York 14620, USA

**Keywords:** Pregnancy, Father involvement, Healthy start and fathers

## Abstract

**Background:**

Defining male involvement during pregnancy is essential for the development of future research and appropriate interventions to optimize services aiming to improve birth outcomes. Study Aim: To define male involvement during pregnancy and obtain community-based recommendations for interventions to improve male involvement during pregnancy.

**Methods:**

We conducted focus groups with mothers and fathers from the National Healthy Start Association program in order to obtain detailed descriptions of male involvement activities, benefits, barriers, and proposed solutions for increasing male involvement during pregnancy. The majority of participants were African American parents.

**Results:**

The involved “male” was identified as either the biological father, or, the current male partner of the pregnant woman. Both men and women described the ideal, involved father or male partner as present, accessible, available, understanding, willing to learn about the pregnancy process and eager to provide emotional, physical and financial support to the woman carrying the child. Women emphasized a sense of “togetherness” during the pregnancy. Suggestions included creating male-targeted prenatal programs, enhancing current interventions targeting females, and increasing healthcare providers’ awareness of the importance of men’s involvement during pregnancy.

**Conclusions:**

Individual, family, community, societal and policy factors play a role in barring or diminishing the involvement of fathers during pregnancy. Future research and interventions should target these factors and their interaction in order to increase fathers’ involvement and thereby improve pregnancy outcomes.

## Background

Paternal involvement (PI) has been recognized to have an impact on pregnancy and infant outcomes [1-6]. When fathers are involved during pregnancy, maternal negative health behaviors diminish and risk of preterm birth, low birth weight and fetal growth restriction is significantly reduced [1-4,6]. PI has also been associated with infant mortality up to one year after birth [2]. When these findings were stratified by race, several studies report that the risks of adverse birth outcomes and subsequent infant mortality were markedly higher for African-American mothers [1,2,4,7].

Whether measured through proxies such as paternal information on birth certificates, maternal report of paternal activities (support, presence at pregnancy-related health appointments), or marital/partnership status, findings point to the important contributions fathers can make to improving birth outcomes [1-4,6-9]. Researchers have proposed that the mechanisms through which PI affects birth outcomes are primarily linked to the impact fathers can have on influencing maternal behaviors and reducing maternal stress through emotional, logistical and financial support [6]. For example, pregnant women with involved partners have been found to be more likely to receive early prenatal care and to reduce cigarette smoking [9,10]. Other studies have suggested that support from fathers serves to alleviate the burden of stress [3] and improves maternal wellbeing, both pathways to improved birth outcomes [11,12].

Despite the evidence that PI during pregnancy is important, there is a dearth or knowledge which continues to hinder progress towards understanding the role of fathers during pregnancy and the subsequent development of appropriate measurements, of policies and of interventions to increase PI during pregnancy [13]. Given the lack of consensus among researchers about what it means to be an “involved father” and to identify the specific roles of a father during pregnancy, this study sought the perceptions of fathers and mothers themselves. Defining and describing male involvement during pregnancy is essential for the development of future research and to inform appropriate interventions to increase male involvement.

## Methods

In an effort to inform next steps for the National Healthy Start Association’s “Male Involvement - Where Dads Matter” initiative, this research project was designed and executed by a workgroup of concerned fathers, mothers, and maternal and child health specialists. Members included community members, grassroots organization leaders, and academic researchers with the same vision of further delving into the issue of the role of fathers during pregnancy in order to better inform initiatives aiming to improve the health of infants. Thus this project is rooted in the principles of community based participatory research, in which key community stakeholders are involved in the formulation of the research question and consequential research execution and program development [14-17]. Furthermore, community based participatory research (CBPR) is action oriented and serves the dual purpose of both a service to the community in which it is being performed and a research endeavor [15,17]. In light of the complex social underpinnings of factors that determine a father’s involvement in his child’s life, CBPR, with is fundamental inclusive approach of both researcher and community stakeholder, was a necessary approach to further delving into the issue.

### Procedures

The research and focus group protocols were developed by the research workgroup and approved by the University of Rochester Medical Center Research Subjects Review Board (RSRB 00036193), to ensure the protection of the subjects participating in this research.

The study population was recruited from the pool of mothers and fathers attending the National Healthy Start Association conference held in Washington, D.C., from March 6—9, 2011. Registered members of the conference were recruited via an electronic mail message describing the study and requesting their participation in focus group discussions. Additional recruitment was conducted at the conference through announcements during plenary sessions. The primary challenge encountered during recruitment was the high no-show rate of males despite initial agreement to participate. To increase male participation, announcements were made during conference sessions to remind males of the opportunity to share their opinions. All participants were required to sign written informed consent as approved by the University of Rochester Research Subjects Review Board. All persons targeted for recruitment and subsequently enrolled were above the age of 21. Participants provided verbal consent and were assured that they were not obligated to join in the discussion and could cease to participate at any point during the focus group. Data collection consisted of 5 focus group discussions, including 1 all female group, 1 all male group, and 3 mixed-gender groups. Additional focus groups were not completed as saturation was reached with the current sample. Most of the participants were African American and parents of 1 to 5 children. Focus groups were conducted by 3 trained lead moderators, audio-recorded and then transcribed for analysis. The primary objective of the focus groups was to obtain mothers’ and fathers’ thoughts on the role of the father during their partner’s pregnancy to inform next steps of the National Healthy Start Association’s fatherhood initiative.

### Focus group protocol

The perceived role of males/fathers/partners within the context of heterosexual relationships was the focus for this study. The protocol included questions regarding: 1) the characteristics of the ideal male/partner/father during pregnancy; 2) definitions (father, male partner, biological father, etc.); 3) specific activities the ideal father might undertake; 4) differences between the ideal and reality; 5) benefits and barriers to paternal involvement; 6) recommendations for addressing those barriers; and 7) additional themes raised by participants. Team members met following the first focus group to discuss the process, content, and any issues that arose. No changes were made to the protocol questions.

### Participants

A total of 50 mothers and fathers participating in National Healthy Start Association pre- and post-natal health services (N = 37 females, N = 13 males) took part in the focus group discussions. The majority were African American (N = 43). Socio-economic data was not collected due to the nature of focus groups. Participants were from one of 102 National Healthy Start Association sites across the United States, providing a range of income and education levels and geographic regions [18]. Although there were 2 gender-specific groups and 3 mixed groups, there was consensus within and between all groups regarding the role of fathers.

### Analysis

**Figure 1:**
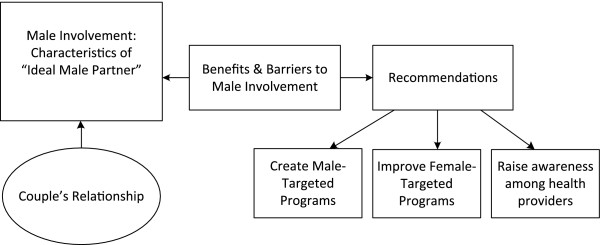
**Focus group data categories.** This flow chart demonstrates the major categories into which the focus group data was demarcated and the relationship between the categories. Categories were derived from the grouping of major themes and constructed via consensus amongst authors and community partners to more efficiently organize the data for analysis.

Content and thematic analyses were conducted from transcriptions of the focus groups recordings. All members of the research group participated in examining and interpreting findings. Content analysis consisted of identifying the particulars: specific roles of fathers/men and specific barriers to involvement. Thematic analysis enabled the extraction of general themes regarding fathers during pregnancy. By definition, a theme is a pattern found in the information that at the minimum describes and organizes possible observations or at the maximum interprets aspects of the phenomenon [19]. Thematic analysis is widely used in qualitative research and is an inductive process based on direct observation of patterns yielded from the interview data particulars in this case and the interpretation of these patterns to more efficiently organize the data [14,19,20]. The themes identified revolve around detailed descriptions of the role of males during pregnancy, concerns regarding male involvement during pregnancy, as well as recommendations for addressing the issues raised (Figure 1). Findings are presented in the following section, and, as much as possible, participants’ words have been retained and presented in italics.

## Results

### Role of male partner vs. biological father

It was initially important to identify the “father” or “male partner” prior to moving forward with descriptions of paternal involvement. Focus group participants recognized that the “ideal” situation is that the “love partner” and the “biological father” are the same person; however, they recognized that this is not always the case. There was a general consensus that, should a man become involved with a pregnant woman, he would also accept partnership in the pregnancy, “fill the gaps” of an uninvolved biological father and support her through the process. Therefore the expectations of a “love partner” are similar to that of the ideal biological father.

“I don’t see any difference, because if you’re gonna be participating, or be a part of the child’s life, and also along with the expectant mother, then you are assuming these roles as a father, a dad, a caregiver.” [Male]

The participants also had clear definitions about the various roles a male can have. A “*Daddy*” was described by the women as “*someone who comes and goes,”* the “*sperm donor,”* or the “*biological father.”* On the other hand, a “*Father*” is the male who nurtures and raises the child, regardless of the biological relationship. Finally, a “Man” is someone who “*steps up*” and raises another man’s child (which they recognized to be a growing trend). Women do not desire a “*Daddy*;” they want someone who will be a “*father*” or a “*man.”* The men agreed with the definition, but insisted that taking care of another man’s child is not to be seen as an obligation or a requirement for being a “man,” but rather as a caring act.

### Characteristics of the “ideal” father

Participants were very specific regarding the characteristics of an “ideal father” during pregnancy and provided details regarding activities demonstrating ideal involvement. The ideal father is *present*, *accessible* and *available*, an active participant during the pregnancy. He is present at prenatal visits, ultrasounds, Lamaze classes, parenting classes, in the delivery room cutting the umbilical cord, and helps with birth-related paperwork.

“He's going to the appointments, and he is accessible and available… When I call, he answers…he comes. And when he's there, he's there. He's present.” [Female]

However, apart from the expectation for the partner to be present, respondents emphasized the need for him to be an active participant in the pregnancy process.

“From going with her to the first ultrasound, the first doctor's visit; each time they go he should be a part of that and encourage her. … not just a standby, but actually a participant.” [Female]

An active father cares about the pregnancy, asks questions of the mother and healthcare provider, and is eager to learn more about the process and what is required for a healthy pregnancy. Further, a father provides *physical* and *emotional support* to the woman carrying his child. He helps the mother make important decisions such as creating a birth plan or choosing a name for their child. He *encourages* the mother and provides *positive affirmation* about her body image and reassures her about her ability to be a good mother. He knows about the changes in her body and *understands* the influence of hormones. He *empathizes* with her and is *patient* with her, remaining calm when her emotions fluctuate. Simply *listening* to her and allowing her to vent provide great emotional relief to the mother.

“His role should be one of an encourager…[Giving] positive affirmation … build her confidence so that she can embrace this new [challenge and that] they can embrace it together as a unit.” [Female]

Both men and women participants emphasized this idea of “*togetherness*” during pregnancy and beyond, which offers great security for the mother. It is the idea that there is an equal investment and interest in having the child, and that the required responsibility of having the child is willingly shared between the two parents. This is important so the woman can feel she is not alone.

“It’s our baby, not just my baby…We have to go through this together and not everything just towards me. Not me just going to the doctors, we need to go through all this together.” [Female]

The ideal father is also a *comforter* and *caregiver*, making certain she feels as comfortable as possible. Examples participants provided include: “*he rubs her feet*,” “*does midnight runs to satisfy her cravings*,” “*takes her shopping and to the movies*.” Respondents also described how the ideal father is *supportive in the physical environment*, the home. He helps with the cooking, cleaning, washing clothes/dishes, and taking care of the other children in the home. They recognized the importance of the father in encouraging healthy behaviors during pregnancy, helping her manage her diet and exercise during the pregnancy: ensuring that she eats a balanced and healthy diet, taking her for walks, exercising with her.

“Making sure she eats healthy, go to her doctor’s appointments. If she has to take off because of a high risk pregnancy, he’ll maintain the bills, or other kids, if there are other kids, just whatever is needed.” [Male]

Interestingly, in all five focus group, financial support was not brought up by male or female participants until the moderator specifically asked about it. This indicates that, contrary to popular belief that men are seen primarily as the financial provider, it was not at the forefront of their thinking about fathers’ role during pregnancy. When asked about it, participants indicated that *financial support* is important, however, emotional and physical support is crucial during the pregnancy period. Ideally, he would help maintain the bills and stay employed. They specified that men should be involved regardless of their ability to provide financial support. Overall, the ideal father is seen as the *protector* who does what is necessary to ensure the safe journey of the baby and the mother.

“The role of a protector is very important, because if he understands how cigarette smoking can impact her, if he’s smoking, then he should understand the impact that it has on wanting to protect both his partner and the unborn child. So I see his role as protecting to ensure a safe journey.” [Male]

To adequately embrace all of these characteristics, respondents felt strongly the ideal father must be *responsible* and *mature.* He must have a sense of responsibility for caring for the child and possess the maturity to carry out the requirements of the role. Here the issue of age comes into play, as participants recognized younger fathers are not always well prepared to handle the emotional responsibility that comes with expecting a baby.

### Defining male involvement

In addition to characterizing the ideal father/partner during pregnancy, participants were asked to define a father’s involvement during the pregnancy period. The following 2 definitions were specifically verbalized at the conclusion of the discussions, with the group members deciding the appropriate wording and agreeing with their final statement:

“The role of a man during pregnancy is to be present, to support, to understand, to be patient, and to have sympathy for the woman carrying his child.”

“The role of a man during pregnancy is to provide emotional, physical and (if possible) financial support to the woman carrying his child.”

### Couple’s relationship

An important concept raised in the focus groups was the “*love relationship*” between the couple - the mother and the father or male partner during pregnancy. This relationship was cited as pivotal in terms of male involvement in the pregnancy. While many women mentioned this as the ideal or preferred situation, male participants also emphasized their relationship to the mother would determine the level of their involvement during the pregnancy process.

A female participant explained: *“If I’m pregnant and I have this significant other,… and I tell them, you know, ‘Be with me,’ that's the main thing, ‘Just be with me’ during this time.”* A male participant resonated the sentiment that the *“man will treat that child in an equal proportion to how he feels about the woman.”*

The emotional or romantic connection (or lack thereof) can heavily influence the level of involvement by the father as well as the mental well being of the mother both during and after pregnancy. The following comment by a male participant explains the dilemma that men face when he fathers a child with a woman whom he feels little emotional connection:

“When my wife was pregnant, I was at all the appointments…so I was there…I think it depends how you feel about that girl…[If] you just got some random chick pregnant you’re not going to feel like going to an appointment with her, you’re not going to feel like, encouraging her. It’s like, ‘Man I can’t believe I got this chick pregnant.’…He needs to find some support for himself to be like, ‘you have to do this’, it has to be some kind of encouragement for him.” [Male]

Being romantically involved with the mother was cited as contributing to her emotional well being and important in relieving the bouts of depression that can occur in pregnancy.

“Since he’s not in the picture, for a woman, automatically, she’s depressed. I don’t know too many women who are pregnant and want their man to not be there. Normally she wants him there and just the near presence of knowing he is still a part reduces your stress level.” [Female]

It also contributed to her feeling of security that this person will be there even after the pregnancy.

“She wants to know that he’s in this as well as she is, and that parenting is a two-way street. It’s not just about mom. And mom needs to know that he’s going to be there afterwards and provide the same love and support that he’s providing when she’s pregnant.” [Female]

During the discussion men and women highlighted that often, the couple’s relationship takes precedent over focus on the child. As one female respondent put it: *“Nobody thinks about that baby. No one thinks about [it being] their pregnancy. All they see is the mom, dad, and the relationship.”* The general consensus was the focus should be primarily on the child, regardless of the status of the couple’s relationship.

In the discussion surrounding the couple’s relationship, participants often stressed the significance of healthy *communication* between parents. Largely they felt that *“communication beforehand,”* or in the early stages of the relationship prior to conception can have important implications to how the pregnancy experience is managed. Another aspect raised is the differences in communication styles. Women felt men often cannot articulate their feelings well and are not as open to sharing [their emotions] thus straining the parental relationship. In contrast, men felt women often were loud and accusatory when they attempted to talked with them.

“Men and women aren’t the same. Men have a difficult time communicating. We don’t communicate well with each other. And we certainly cannot express feelings.” [Male]

### Benefits of fathers’ involvement

The primary benefits of having a father or male partner involved during pregnancy were the reduction of maternal stress levels and the encouragement of positive maternal behaviors. Participants believed these to have implications for the health of the baby. They reasoned that if the mother’s physical and mental health was optimal, then the benefits would be observed in the baby.

“Be agreeable, take less stress off of her cause by her being an African black woman, infant mortality rates is high, and if you put all this stress on her, it’s putting stress on the baby.” [Female]

Indeed, African American participants in particular were very aware of the higher rates of infant mortality within their community compared with others. They strongly felt that if fathers would be more involved and help reduce maternal stress levels, it would positively impact infant outcomes. Participants expressed that African American mothers as a group are more vulnerable to lack of PI:

“I think particularly in African-American community we have too many single parents. We have far too many. Not only that, the divorce rate is high in African-Americans too so we need to establish strong relationships and build stronger marriages.” [Female]

### Barriers to fathers’ involvement

**Figure 2:**
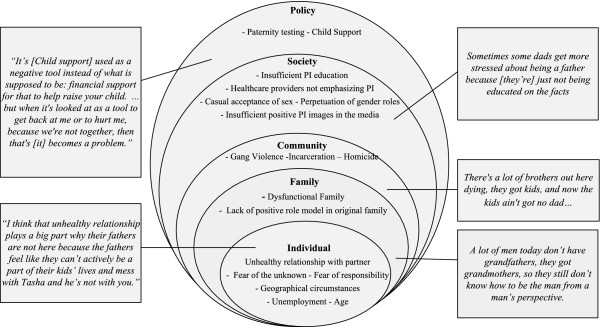
**Barriers to fathers’ involvement during pregnancy.** In this figure we dissect the barriers to father’s involvement by levels in the socioecological model. Within in each sphere of influence starting from the individual and extending out towards the policy level, detail is provided about the potential barriers categorized at this level as revealed in the focus group data. Actual quotes from participants are inserted at each level to provide examples.

Participants recognized that there are sadly, many barriers to fathers’ involvement during pregnancy. These can be broadly categorized using Bronfenbrenner’s socioecological model [21] (Figure 2).

As displayed in Figure 2 participants indicated that the major deterrents to father’s involvement at the individual level are: an unhealthy relationship between the father/male partner and the biological mother, and men in general not knowing their role or not wanting the new responsibilities (financial and time) associated with having a child. They attribute this largely to general lack of education available for expecting men, lack of positive role models in the family sphere, and dysfunctional family foundations. The expense and insufficient availability of paternity testing can also be a deterrent for involvement if the man questions the legitimacy of the child being his. A lack of understanding of the role of Child Support and lack of knowledge of regulations and legal financial responsibilities were also cited as significant obstacles to men’s involvement.

Participants also bemoaned the popular public acceptance of casual sexual encounters which belittles the role of parenting. Participants felt that males do not adequately prepare for the possibility of pregnancy. Furthermore, they believed that societal, perpetuation of gender roles places the responsibility of caring for children on women. Men are expected to provide financially, but media based stereotypes, are not obligated to provide emotional or physical support. Participants wished examples of men being good fathers were better marketed. In addition, participants felt that social problems such as involvement in gangs and violence contributed to absent fathers through incarceration or homicide.

Of note, participants commented on the importance of the relationship of the father and the mother’s family. Specifically, respondents felt that if the mother of the mother, i.e. the grandmother, fostered an accepting relationship with the biological father he would be more apt to be involved in taking care of the mother and the coming child. This is in line with family systems theory, which posits that cross generational triangulation patterns can reverberate across generations [22].

In sum, participants viewed involvement of fathers during pregnancy time as very positive for the mother and the child. However, they also recognized many prevailing barriers to a man fully realizing his role as an expectant father.

### Community recommendations for increasing fathers’ involvement during pregnancy

**Figure 3:**
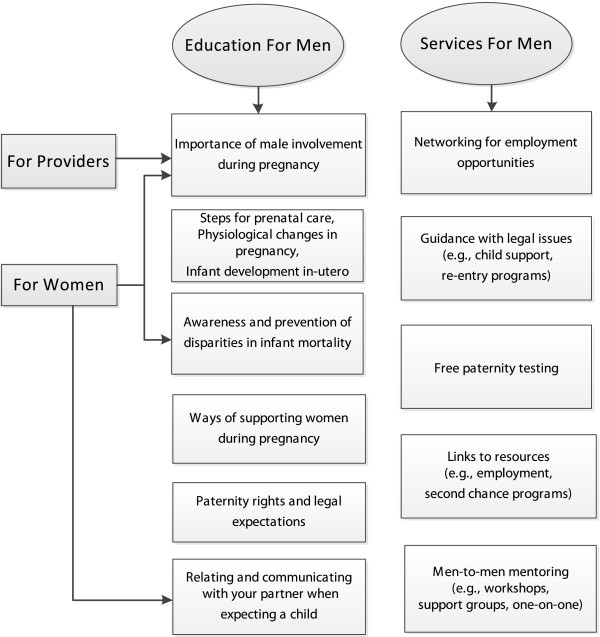
**Recommendations for programs aimed at improving pregnancy outcomes by increasing male involvement.** Recommendations were provided throughout the focus group data and focused mainly on education for men and the services that should be provided to promote ideal paternal involvement. Included were suggestions for education and/or services targeting women and service providers.

Participants identified a number of strategies that could increase the involvement of men during pregnancy. Figure 3 displays these recommendations by target populations including women, men and health care providers. Primarily, participants were unanimous that there needed to be education for men to increase their knowledge of paternity rights and expectations, as well as the pregnancy process. Alongside educational efforts, participants desired interventions that provided men with links to vital resources (paternity testing, information on child support regulations, second chance programs) and employment opportunities, especially for those with a disability or previous incarceration. Participants also emphasized that health care providers and women also need to appreciate the involvement of men during the prenatal period and its impact on maternal and child health. Finally, participants recognized the importance of providing support and training to men and women, particularly communication skills, that might strengthen their relationship as it pertains to the child’s needs.

## Discussion

**Figure 4:**
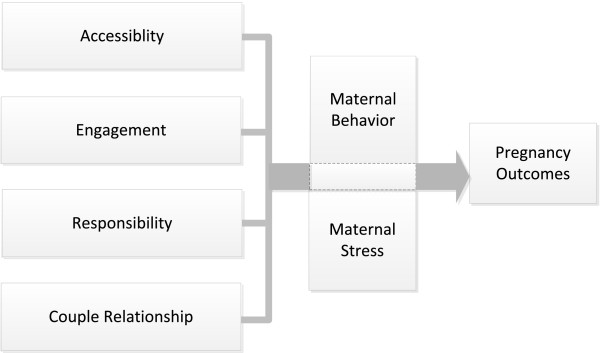
**Model for fathers’ involvement during pregnancy.** The model conceptualizes the 4 components of a proposed framework for paternal involvement during pregnancy. The model reflects similar elements to Lamb’s theory of paternal involvement in childhood (accessibility, responsibility, engagement). In pregnancy, the extent of these fundamental characteristics is mediated by the nature of the relationship between the mother and the male involved.

In this study, an involved man or father during pregnancy is defined by participants as being accessible (e.g., present, available), engaged (e.g., cares about the pregnancy and the coming child, wants to learn more about the process), responsible (e.g., is a caregiver, provider, protector), and maintaining a relationship with the woman carrying the child, regardless of their own partnership status. This is partly in line with findings on involvement of fathers with children, particularly Lamb’s theory which posits three components, engagement, accessibility, and responsibility, which associate moderately with each other and determine the degree of involvement of the man [23]. These dimensions refer to father-child relationships. Based on this study’s findings we expand this theory in the context of pregnancy, riding on the assumption that both the mother’s and the father’s role began with insemination, through pregnancy and continues after the birth of the child. These concepts would then be applied to the father in relation to the mother carrying the child. Paternal involvement during pregnancy, though similar to paternal involvement during childhood, has a specific difference which we identify as the relationship between the father and the mother carrying the child. We therefore propose that paternal involvement during pregnancy is composed of 4 elements (accessibility, engagement, responsibility, and couple relationship) which may lead to positive pregnancy outcomes through the reduction of maternal stress [3] and support for positive maternal behaviors [6,9] (Figure 4). The couple relationship element is intertwined within each of the first three components of paternal involvement during pregnancy.

### Accessibility

Participants honed in on the terms *present, accessible* and *available* to describe one level of male involvement during pregnancy, which aptly encapsulates the concept of Accessibility referred to by researchers in the field of childhood development [23-25]. During childhood, accessibility refers to the father being physically present and available to supervise the child, but not actively participating in the same activities as the child. For instance the father may be at the playground observing the child, but not playing with the child [23,25]. In a similar vane, our study participants felt that the father’s physical presence both in the home (ideally) and at prenatal activities is associated with achieving the perception of parental “togetherness” in the pregnancy process. However the father’s willingness and/or ability to embrace this notion of Accessibility is heavily dependent upon his relationship with the mother. Similar to parents raising a child, the quality of the parental relationship often impacts subsequent involvement with the child [26-28]. Communication between parents, whether prenatally or postnatally, is important to ensuring that fathers are involved. This becomes even more crucial when parents are not in the same household. Research on divorced parents, shows that fathers’ postnatal involvement with their children diminishes greatly due to breakdown in the parental relationship, poor communication [29] and loss of paternal satisfaction [30,31].

### Engagement

The second component of the framework for fathers’ involvement in childhood is engagement. In infancy, engagement is the direct interaction of the father with the child, playing with him, reading books to the child etc. [23-25]. In pregnancy however, this interaction is directed towards the mother and requires active participation in prenatal activities (e.g. reading prenatal care books and asking questions at prenatal care visits). Analogous to paternal involvement in child rearing, in pregnancy the interplay between Accessibility and Engagement is a rhetorical relationship. As the father avails himself to the mother by being physically present, the expectation for him to also be an active participant or actively engaged in the prenatal process becomes evident. Lamb’s framework suggests that accessibility without the ensuing components of engagement and responsibility constitutes an incomplete structure to paternal involvement that will deprive the child of critical support needed for healthy development and emotional well being [23,32]. Pregnancy parallels this as it has been indicated that the lack of male involvement is strongly correlated with higher infant morbidity and mortality [6,33,34], reinforcing the need for effective strategies that seek to increase men’s involvement prenatally. However, as our study confirms, the degree of involvement is proportional to the quality of the relationship with the mother and the parent’s desire for the pregnancy [9,27,34,35]. This is consistent with our model that places Engagement in the context of the couple’s relationship.

### Responsibility

Responsibility embodies the multifaceted roles that fathers/male partners play in financially supporting the child from birth [23,25]. However, prenatally, this responsibility towards the coming child is directed towards the mother carrying the child and extends beyond finances. In our study the concept of Responsibility was manifested in the father assuming the roles of caregiver, provider, nurturer and protector. Community members recognized that men’s involvement is a protective factor that helps to ease maternal stress and to encourage positive maternal behaviors. This has been demonstrated in the literature on pregnancy outcomes. Women whose partners were involved in their pregnancy were more likely to receive prenatal care [9,34], and less likely to give birth to low birth weight and premature infants [33,34] and to promote positive maternal behaviors [9]. Although fathers’ involvement has been deemed difficult to measure, there is consensus that women with more support from their partners tend to have healthier babies. Similar to Accessibility and Engagement, Responsibility is embedded in the context of the parental relationship since discordance in the parental rapport can impinge on the father’s willingness or ability to act responsibly.

Many other socio cultural factors may impact fathers’ involvement during pregnancy. As highlighted in our study, lack of substantive preconception and prenatal education for men, consistent undervaluing of fathers’ involvement by health care providers, and dysfunction in the fathers’ (or mother’s) family of origin often lead to ambiguity regarding their role as fathers and its significance [27,36]. The predominance of social messages that highlight the mother’s role in child rearing while negating that of the father also compounds the issue of father absenteeism or disillusionment [27,37]. Further, social media images often promote casual sexual encounters without adequate contraceptive messages or messages regarding paternal responsibility in the event of an unintended pregnancy; this creates an atmosphere that can increase the occurrence of pregnancy (in adolescents) [38] as an unplanned and untimely event, which has been shown to decrease the level of fathers’ involvement [9,34].

#### Strength and limitations

To our knowledge, this is the first study that attempts to characterize fathers’ involvement during pregnancy, apply theoretical models to understanding the results, and propose a new model for further testing. The use of community based participatory research to develop and implement the research project is an important aspect that lends additional credence to the study findings. This study is a crucial first step for developing measures of paternal involvement and for developing programs to increase fathers’ involvement during this crucial time for infants, *in-utero*. A limitation of the study is that participants were limited to those who attended the National Healthy Start Association conference. Of note is that funding was given across sites to help ensure that those who could not afford it would still be able to participate, ensuring representation across sites as well as across socio-economic status.

Initial data on fatherhood during pregnancy can only be gathered through in-depth conversations with parents and those involved in the day-to-day work with families. Our study provides the basis for larger studies that will take a broader and more holistic approach to family health, regardless of the composition of the “family,” in order to improve health outcomes for mothers and infants.

#### Recommendations

In light of the impact of fathers’ involvement on maternal and infant health, as identified in the scientific research as well as this community investigation, it is crucial that interventions and policies to improve infant outcomes prioritize fathers’ involvement [34,36,39]. Of great importance is the training of health care providers to stress the need to encourage and welcome fathers in the prenatal process, as they are in a key position to be influential to the mother’s health behavior [40]. However males must first be educated about specific expectations of a father, the importance of his role to healthy child development and how he can best support the mother to improve pregnancy outcomes [36]. Information on biological changes in the mother and issues surrounding risk factors for infant mortality would also be integral components of any proposed educational curriculum. To have greater impact, this education process should begin prior to conception and should be targeted specifically to males, addressing their distinctive concerns [26,36,41]. Furthermore, though there are existing parenting programs that provide support for new parents, these often lack the comprehensiveness of co-parent counseling support, male only counseling, resource support (e.g. employment opportunities) [26], and guidance in legal issues such as child support and paternity testing [41]; all of which are urgently needed. A one-on-one male mentoring approach would also be helpful in teaching males how best to fulfill their role, as this community group has highlighted. Improving existing prenatal services for women should also be modified to include information on the importance of paternal involvement to infant health outcomes. Importantly, improved communication skills are crucial to developing better relationships between parents and facilitate increased male involvement to benefit the child.

## Conclusions

Paternal involvement is as crucial prenatally as it has been shown to be postnatally for infants. Fathers are to be accessible and engaged during the pregnancy and begin to demonstrate responsibility towards the coming child by helping the mother. Because all of the involvement is through the mother carrying the child, the relationship between the two parents is of utmost importance and determines the level of involvement. Of note, is that while in the postnatal phase, the financial ability of the father is of paramount importance [26,32], the financial support appears to be much less emphasized during pregnancy when compared with emotional and physical support. Instruments to assess father involvement during pregnancy should include a measure of parental relationship, just as interventions to increase fathers’ involvement must plan for addressing relational factors between parents regardless of their marital status.

Despite participants’ understanding of the importance of PI for maternal health and behavior as well as infant birth outcomes, many barriers to optimal involvement were identified. Individual, family, community, societal and policy factors play a role in barring or diminishing the involvement of fathers during pregnancy. Future research and interventions should target these factors and their interaction in order to increase fathers’ involvement and thereby improve pregnancy outcomes.

## Competing interests

The authors of this manuscript have no significant competing financial, professional or personal interests that might have influenced the performance or presentation of the work described in this manuscript.

## Authors’ contributions

APA designed and implemented the study, conducted the qualitative analyses and was the primary contributor to manuscript development. CL shared in the preparation of the manuscript with Dr. APA and also conducted the qualitative analyses. KS and KH participated in the study design, implementation and data collection, reviewed manuscript and made revisions to ensure that community interests were well represented. KH reviewed and made edits of the manuscript to ensure that the content and structure was in keeping with National Healthy Start objectives. KF reviewed the manuscript for accuracy and goals of research project. All authors read and approved the final manuscript.

## Pre-publication history

The pre-publication history for this paper can be accessed here:

http://www.biomedcentral.com/1471-2393/13/60/prepub
